# Supplementary material for: Algal cell bionics as a step towards photosynthesis-independent hydrogen production
Source: Nat Commun. 2023 Apr 4;14:1872. doi: 10.1038/s41467-023-37608-4 (PMC10073198; doi:10.1038/s41467-023-37608-4)
Supplement: Supplementary file 1 — Supplementary Information [file 41467_2023_37608_MOESM1_ESM.pdf]

Algal cell bionics as a step towards photosynthesis-independent hydrogen production

Xu *et al.*

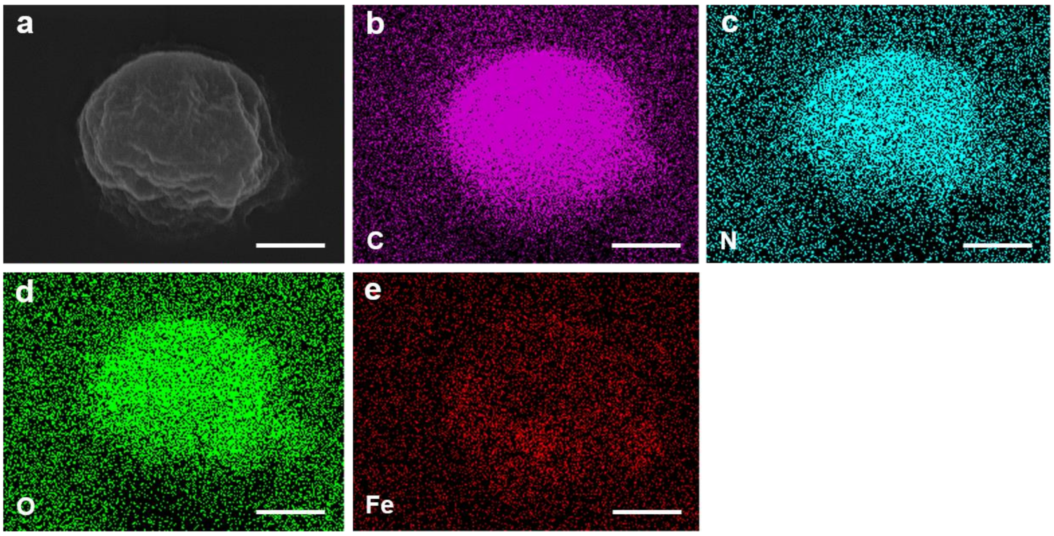

Supplementary Figure 1. Surface elemental characterization of PPy-coated *Chlorella* cells. (a) SEM image of a single PPy-coated *Chlorella* cell. (b-e) EDS elemental mappings of C (b), N (c), O (d) and Fe (e) on the surface of the single cell shown in (a). Scale bars, 1 μm. All relevant experiments were performed independently at least three times with similar results. Iron is homogeneously distributed on the individual algal cell.

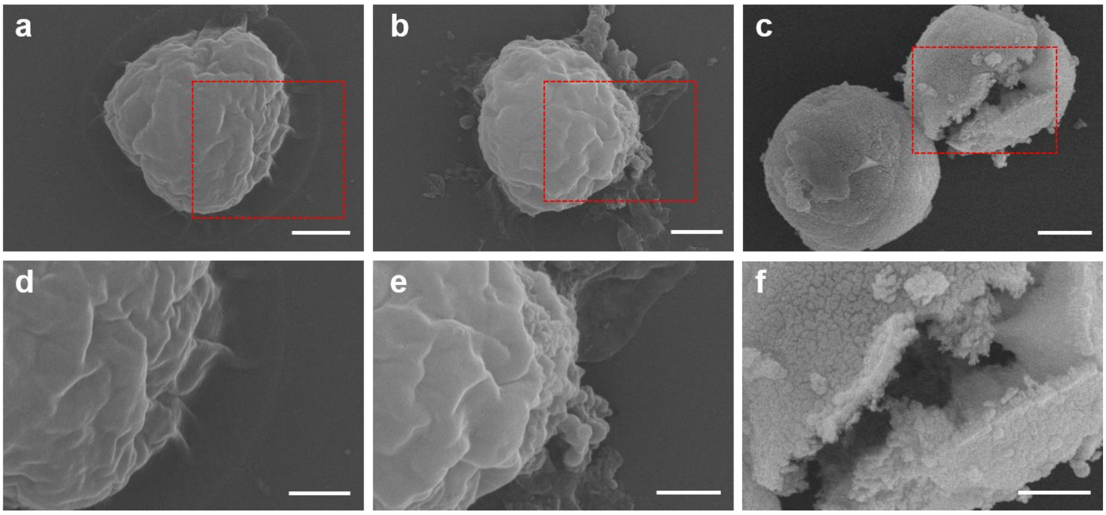

Supplementary Figure 2. Morphology characterization of the native and coated *Chlorella* cells. (a-c) SEM images of whole native (a), PPy-coated (b) and PPy/$CaCO_3$-coated (c) *Chlorella* cells. Scale bars, 1 μm. (d-f) Partially enlarged images of native (d), PPy-coated (e) and PPy/$CaCO_3$-coated (f) *Chlorella* cells (see red frames in (a-c)). Scale bars, 500 nm. All relevant experiments were performed independently at least three times with similar results. Coating of PPy onto the native algal cell increases the surface roughness. The outer coating of $CaCO_3$ produces a continuous rigid endoskeleton around the algal cell.

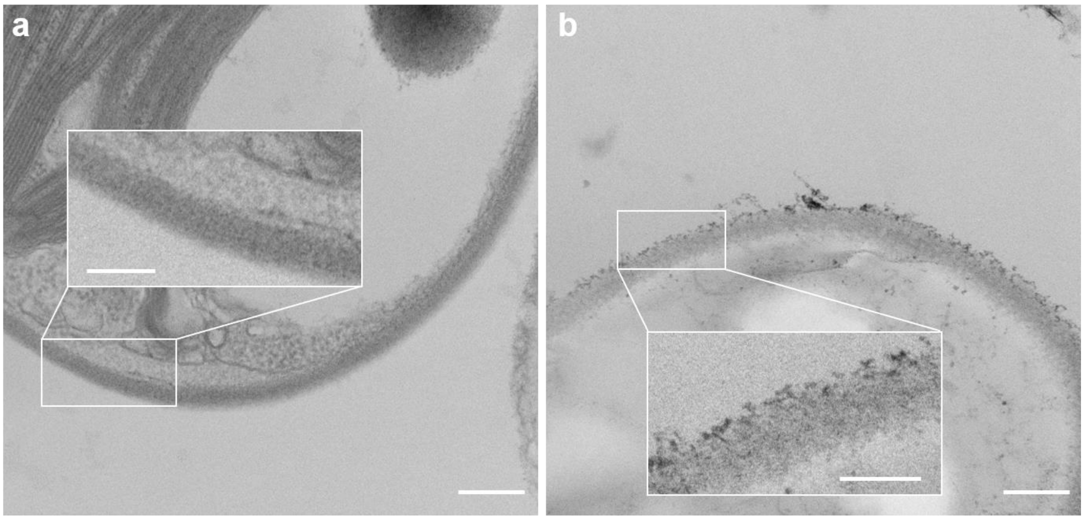

Supplementary Figure 3. TEM images of ultrathin cross-sections for native (a) and PPy-coated (b) *Chlorella* cells. Insets show enlarged images. Scale bars, 200 and 100 nm (insets). All relevant experiments were performed independently at least three times with similar results. While the native algal cell wall is smooth, surface roughening due to the presence of attached PPy nanoparticles is observed for PPy-coated cell.

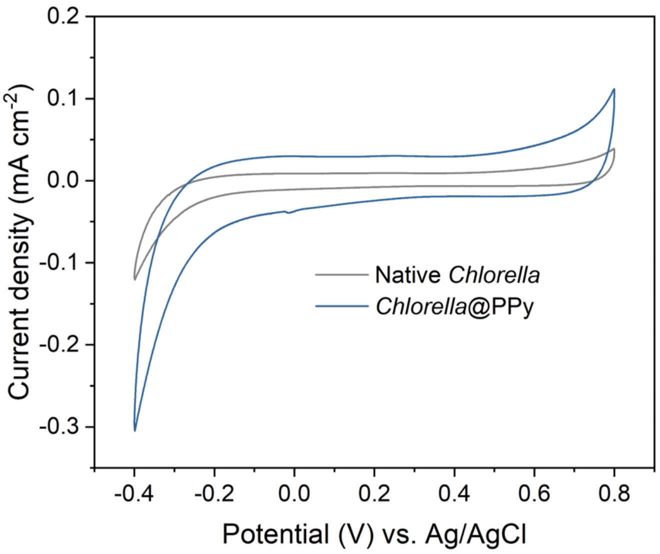

Supplementary Figure 4. CV curves of the native and PPy-coated *Chlorella* cells. The increased current density in the plot for the PPy-coated algal cells indicates the polymerization of pyrrole on the algal cell wall.

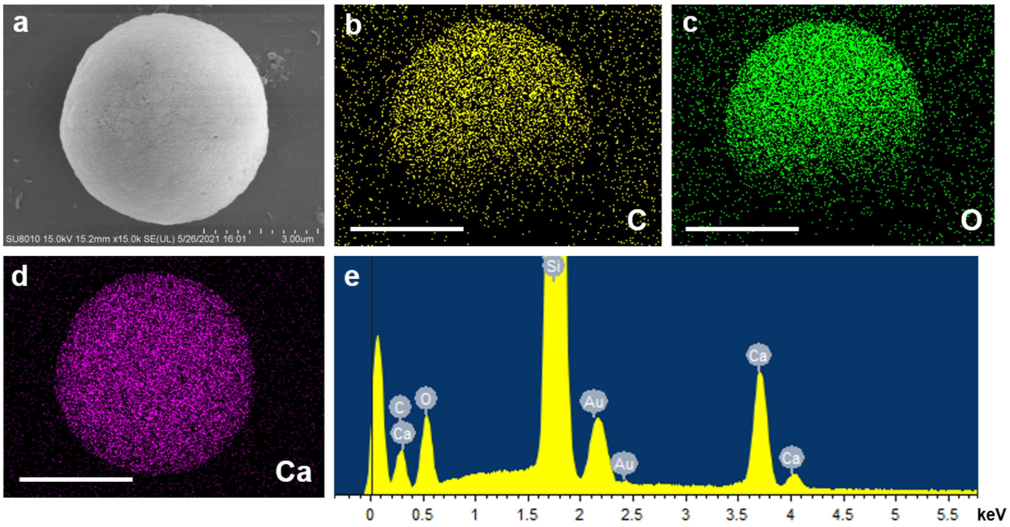

Supplementary Figure 5. Surface elemental characterization of PPy/$CaCO_3$-coated *Chlorella* cells. (a) SEM image of a single PPy/$CaCO_3$-coated *Chlorella* cell. (b-d) EDS elemental mappings of C (b), O (c) and Ca (d) on the surface of the single cell shown in (a). Scale bars, 3 µm. (e) Corresponding EDS elemental spectra showing the element distribution on the cellular surface. All relevant experiments were performed independently at least three times with similar results. C, O and Ca are present in the outer shell. The Si signal is from the substrate and Au is used as a conductive sputter coating.

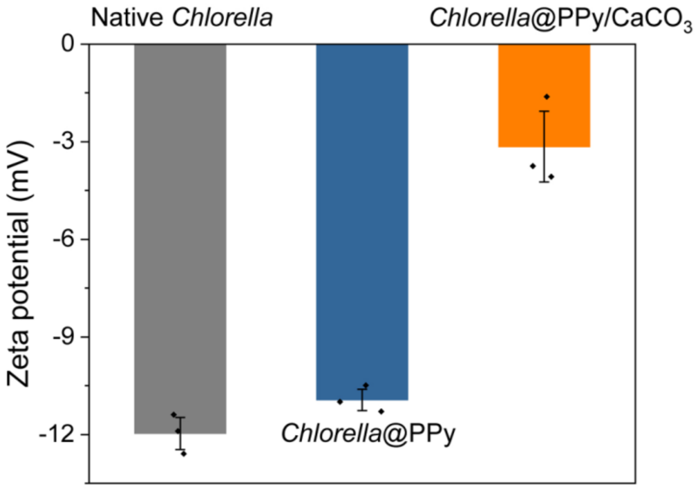

Supplementary Figure 6. Zeta potential measurements of native, PPy-coated and PPy/$CaCO_3$-coated *Chlorella* cells in PBS buffer (pH = 7.0). Data are presented as mean values ± SD, error bars indicate standard deviations (n = 3, biologically independent samples). Native *Chlorella* cells, -11.97 mV; PPy-coated *Chlorella* cells, -10.93 mV; PPy/$CaCO_3$-coated *Chlorella* cells, -3.15 mV. A slight decrease of zeta potential is observed for PPy-coated cells, probably due to Fe(III) cations on the polymer-coated cell wall. In contrast, charge interactions with Ca^{2+} ions and formation of $CaCO_3$ shell result in a significant decrease in zeta potential. Source data are provided as a Source Data file.

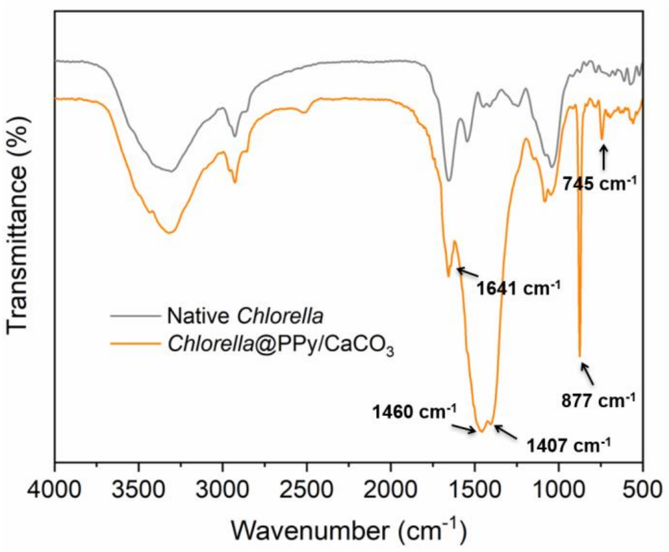

Supplementary Figure 7. FTIR spectra of the native and PPy/$CaCO_3$-coated *Chlorella* cells. The spectra of PPy/$CaCO_3$-coated cells exhibits peaks at 1641 cm^{-1} which are assigned to the conjugated C=C bond in the PPy ring. Peaks at 1460 and 1407 cm^{-1} correspond to the split asymmetric stretch of CO_3^{2-}, and those at 877 and 745 cm^{-1}, correspond to out-of-plane bending of CO_3^{2-} in the vaterite phase of $CaCO_3$. The results indicate successful coating of PPy and $CaCO_3$ shells onto the cell surface.

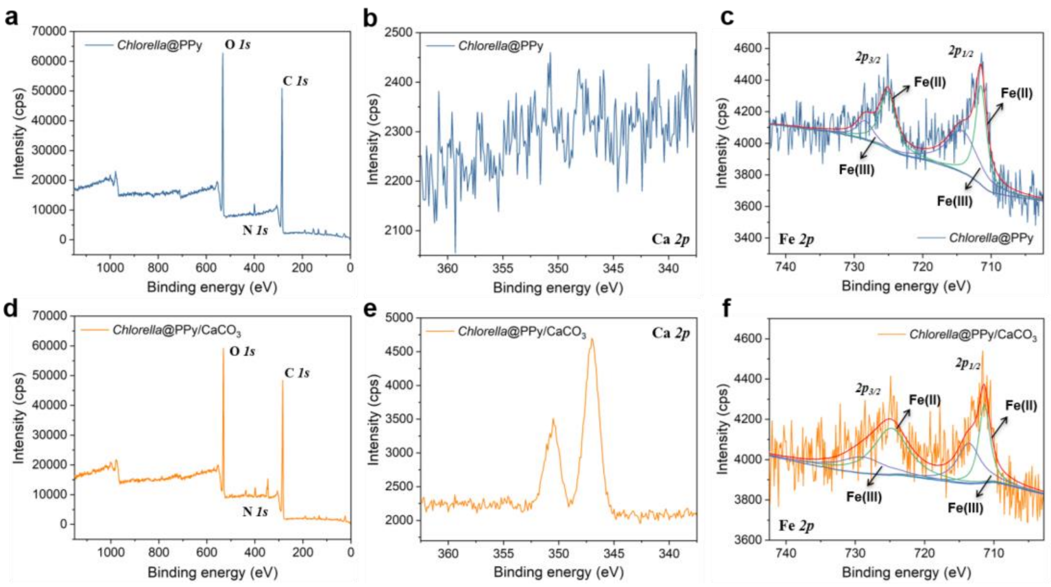

Supplementary Figure 8. Elemental analysis for the coated *Chlorella* cells. XPS spectra of PPy-coated (a-c) and PPy/$CaCO_3$-coated *Chlorella* cells (d-f). Peaks for Fe but not Ca are observed for PPy-coated *Chlorella* cells, indicating the presence of a Fe(III)-doped PPy layer. Strong peaks for Fe and Ca are observed for the PPy/$CaCO_3$-coated *Chlorella* cells.

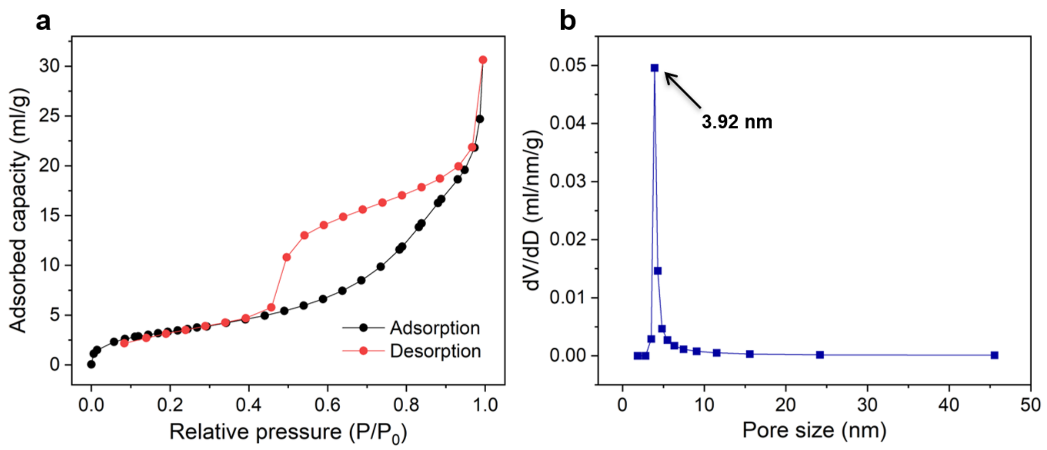

Supplementary Figure 9. Nitrogen adsorption-desorption isotherms (a) and BJH pore size distribution curve (b) determined from PPy/$CaCO_3$-coated algal cells. The average pore size of PPy/$CaCO_3$ shell is 3.92 nm.

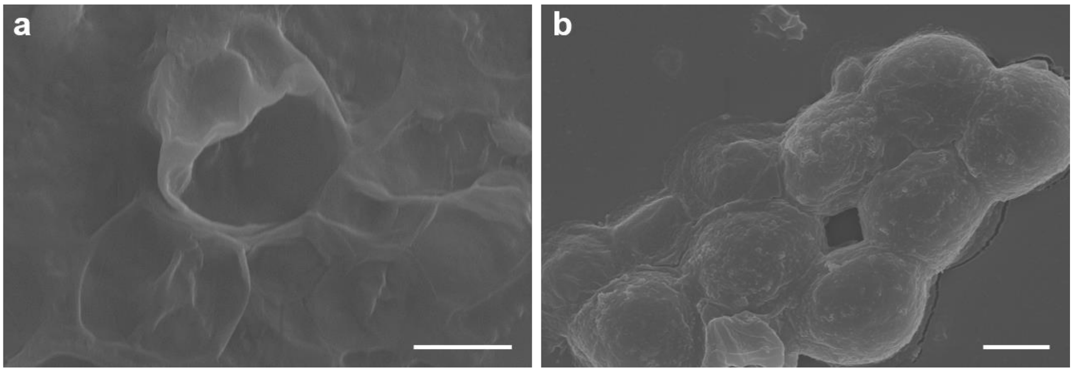

Supplementary Figure 10. Structural stability characterization of the native and PPy/$CaCO_3$-coated *Chlorella* cells under long-term incubation. SEM images of native (a) and PPy/$CaCO_3$-coated (b) *Chlorella* cells cultured in sodium ascorbate-containing TAP culture medium for 7 months. Scale bars, 2 μm. All relevant experiments were performed independently at least three times with similar results. The native *Chlorella* cells collapse due to the leakage of cytoplasm. In contrast, the ellipsoid morphology of the PPy/$CaCO_3$-coated cells is well-maintained, demonstrating the protective role of the $CaCO_3$ shell.

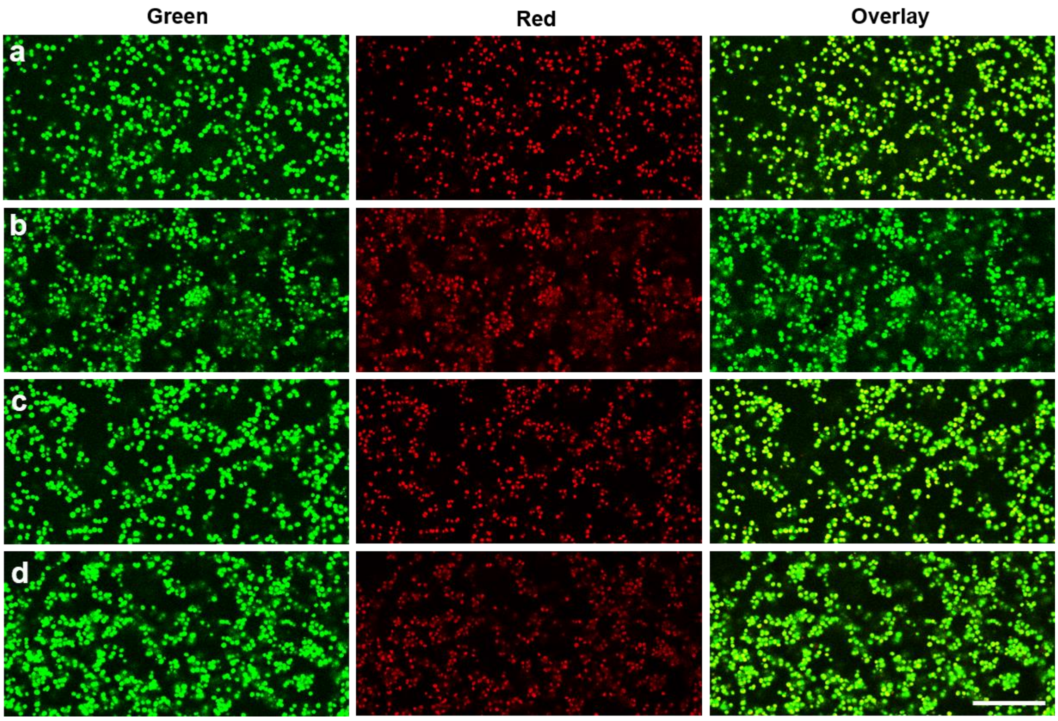

Supplementary Figure 11. Biocompatibility analysis of Fe^{3+} towards living *Chlorella* cell. Confocal fluorescence microscopy images showing the viability of native *Chlorella* cells under the action of Fe(III) ions with concentrations of 0 (a), 0.25 (b), 0.50 (c) and 1.00 (d) mg/mL in DI water for 30 min. Green fluorescence is from fluorescein in viable cells labelled with fluorescein diacetate (FDA), red fluorescence is from intracellular chlorophyll, and yellow fluorescence is from the overlay of the green and red fluorescence. Scale bar, 50 μm. All relevant experiments were performed independently at least three times with similar results. The results indicate that Fe(III) has negligible negative impact on algal cell viability.

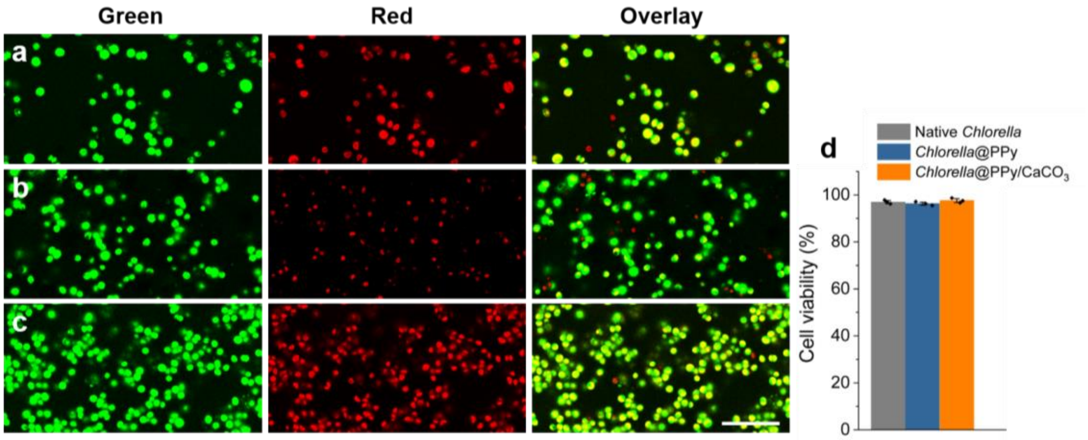

Supplementary Figure 12. Biocompatibility analysis of PPy and $CaCO_3$ shell towards living *Chlorella* cell. (a-c) Confocal fluorescence microscopy images showing the viability of native *Chlorella* cells (a), PPy-coated (b) and PPy/$CaCO_3$-coated (c) *Chlorella* cells. Green fluorescence is from fluorescein in viable cells labelled with FDA, red fluorescence from intracellular chlorophyll, and yellow fluorescence is from the overlay of the green and red fluorescence. Scale bar, 50 μm. (d) Cell viability measurements of native, PPy-coated and PPy/$CaCO_3$-coated *Chlorella* cells. Data are presented as mean values ± SD, error bars indicate standard deviations (n = 3, biologically independent samples). All relevant experiments were performed independently at least three times with similar results. The results indicate that the sequential encapsulation of PPy and $CaCO_3$ impose negligible negative influence on algal cell viability. Source data are provided as a Source Data file.

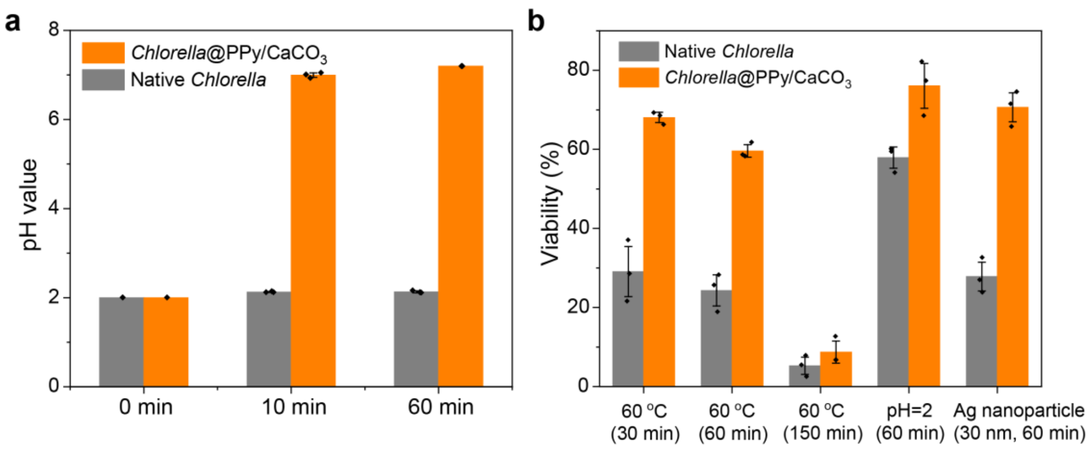

Supplementary Figure 13. **Protective role of hybrid PPy/$CaCO_3$ shell for *Chlorella* cells under adverse conditions.** (a) Time-dependent measurements of pH for native and PPy/$CaCO_3$-coated *Chlorella* cells in acid solution (pH = 2). (b) Cell viability measurements of native and PPy/$CaCO_3$-coated *Chlorella* cells exposed to 60 ºC at neutral pH within various periods, at pH 2 for 60 min, or in the presence of toxic Ag nanoparticles at room temperature and neutral pH. For all experiments, the cells (OD_{750} = 3.0) are immersed in 2 mL of DI water, except in the acid-protective experiment where the solution is replaced by hydrochloric acid (pH = 2). For the Ag nanoparticle-protective experiment, 100 μL of Ag nanoparticle (~0.75 A_{520} units/mL, dispersed in sodium citrate) is added. Data are presented as mean values ± SD, error bars indicate standard deviations (n = 3, biologically independent samples). Coating with a PPy/$CaCO_3$ hybrid layer increases the integrity of the cellular membrane under adverse conditions, thus avoiding the leakage of cellular components under high temperature and increasing cell viability. In addition, the outer $CaCO_3$ layer serves as a sacrificial shell to buffer the acidic environment, which increases the pH to around 7.0 within 10 min, thus prolonging cellular viability. Source data are provided as a Source Data file.

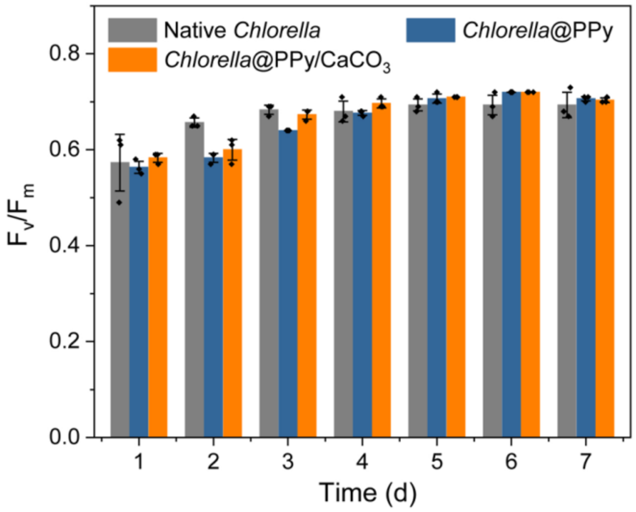

Supplementary Figure 14. Time-dependent measurements of F_v/F_m for the native, PPy-coated and PPy/$CaCO_3$-coated *Chlorella* cells dispersed in pure TAP culture medium. Samples are in sealed vials and exposed to daylight with an intensity of 65 μE m^{-2} s^{-1}. Data are presented as mean values ± SD, error bars indicate standard deviations (n = 3, biologically independent samples). The F_v/F_m of PPy-coated and PPy/$CaCO_3$-coated cells are comparable to that of native *Chlorella* cells, indicating the PPy and $CaCO_3$ coatings have no negative impact on the photosynthetic activity of the algal cells. Source data are provided as a Source Data file.

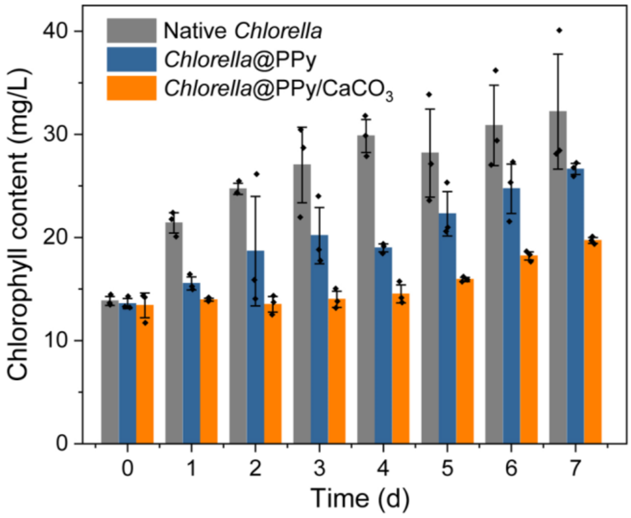

Supplementary Figure 15. Time-dependent measurements of chlorophyll content of native, PPy-coated and PPy/$CaCO_3$-coated *Chlorella* cells cultivated in sodium ascorbate-containing TAP culture medium. Samples are in sealed vials and exposed to daylight with an intensity of 65 μE m^{-2} s^{-1}. Data are presented as mean values ± SD, error bars indicate standard deviations (n = 3, biologically independent samples). The reduced rate of increase in chlorophyll content indicates that the rigid outer $CaCO_3$ layer limits proliferation of the coated *Chlorella* cells. Source data are provided as a Source Data file.

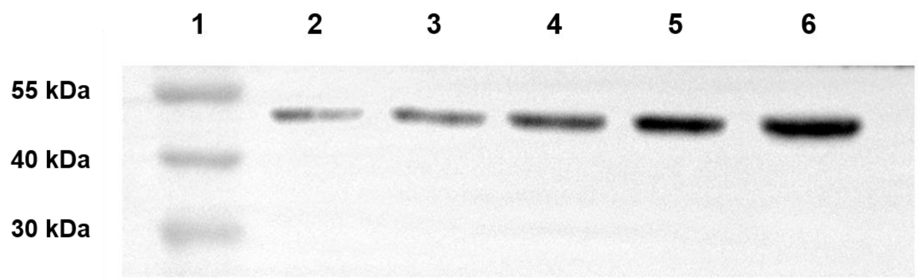

Supplementary Figure 16. HYDA Western Blot analysis of native *Chlorella* cells in normal TAP culture medium and PPy/$CaCO_3$-coated *Chlorella* cells in ascorbate-containing TAP culture medium at different time points. Lane 1: marker; Lane 2: native *Chlorella* cells in normal TAP; Lane 3, 4, 5, 6: PPy/$CaCO_3$-coated *Chlorella* cells in ascorbate-containing TAP culture medium at the time points of 1, 3, 5, 7 d. All relevant experiments were performed independently at least three times with similar results. The time-dependent deepening of band color indicates the gradual expression and activation of hydrogenase in the anaerobic environment induced by the Fe(III)-mediated oxidation of ascorbate. Source data are provided as a Source Data file.

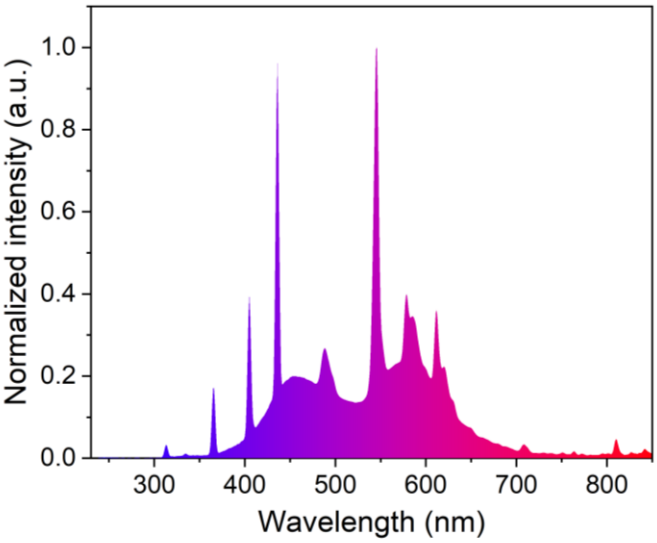

Supplementary Figure 17. The spectrum of light source of used white fluorescent lamp (104 W).

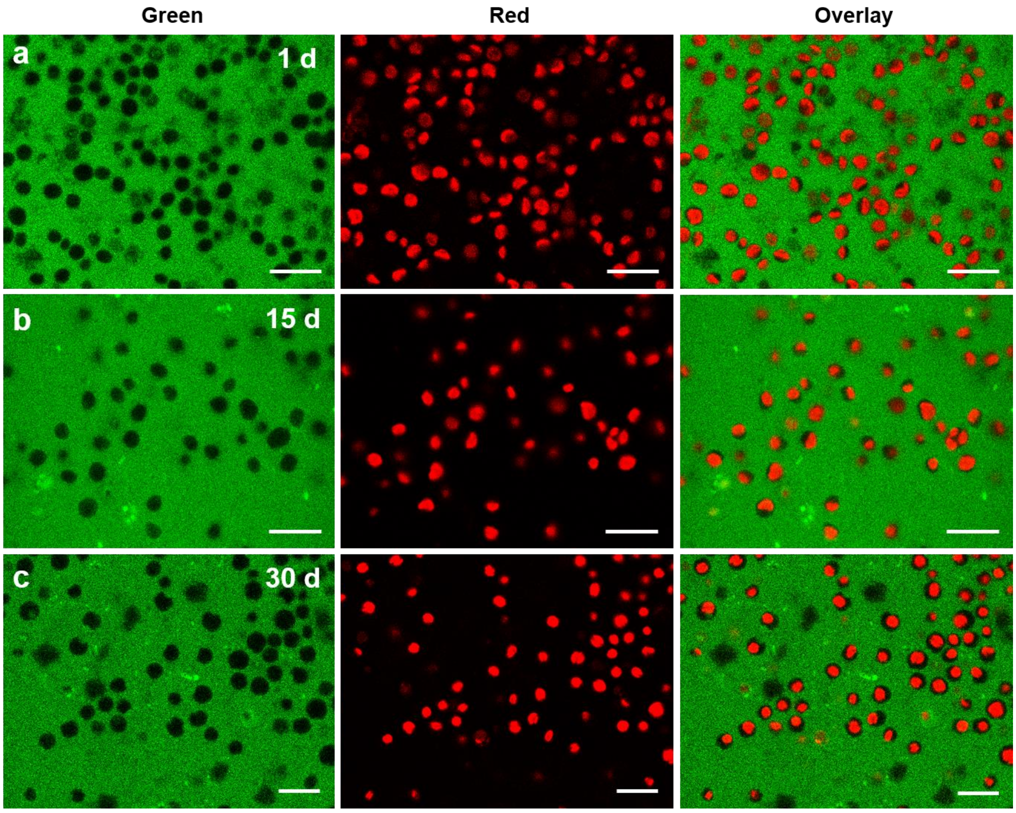

Supplementary Figure 18. Position characterization of EY during the artificial electron-promoted hydrogen production. Time-dependent confocal fluorescence microscopy images of PPy/$CaCO_3$-coated *Chlorella* cells in an EY/TEOA mixture for 1 (a), 15 (b) and 30 (c) days. The algal cells are cultivated in sodium ascorbate-containing TAP culture medium. Green fluorescence is from EY and red fluorescence from the intracellular chlorophyll of the algal cells. Scale bars, 10 μm. All relevant experiments were performed independently at least three times with similar results. EY is located extracellularly, indicating that the exogenous electrons are generated extracellularly during this period.

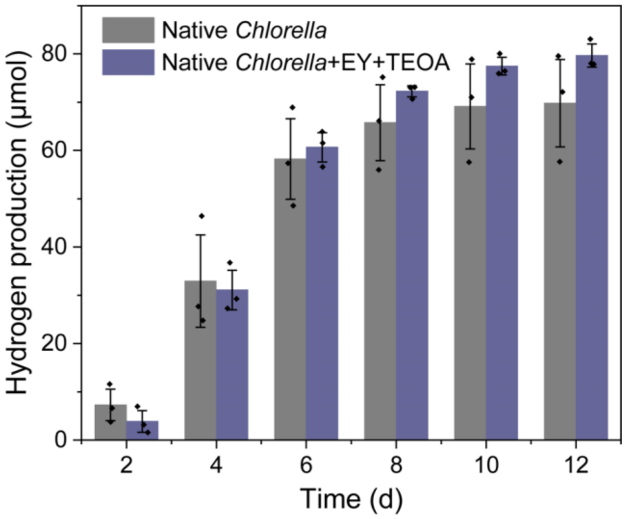

Supplementary Figure 19. Time-dependent measurements of hydrogen production for the native *Chlorella* cells with or without EY and TEOA. Samples are cultivated in sealed vials with sodium ascorbate-containing TAP culture medium and exposed to daylight with an intensity of 65 μE m^{-2} s^{-1}. Data are presented as mean values ± SD, error bars indicate standard deviations (n = 3, biologically independent samples). Source data are provided as a Source Data file.

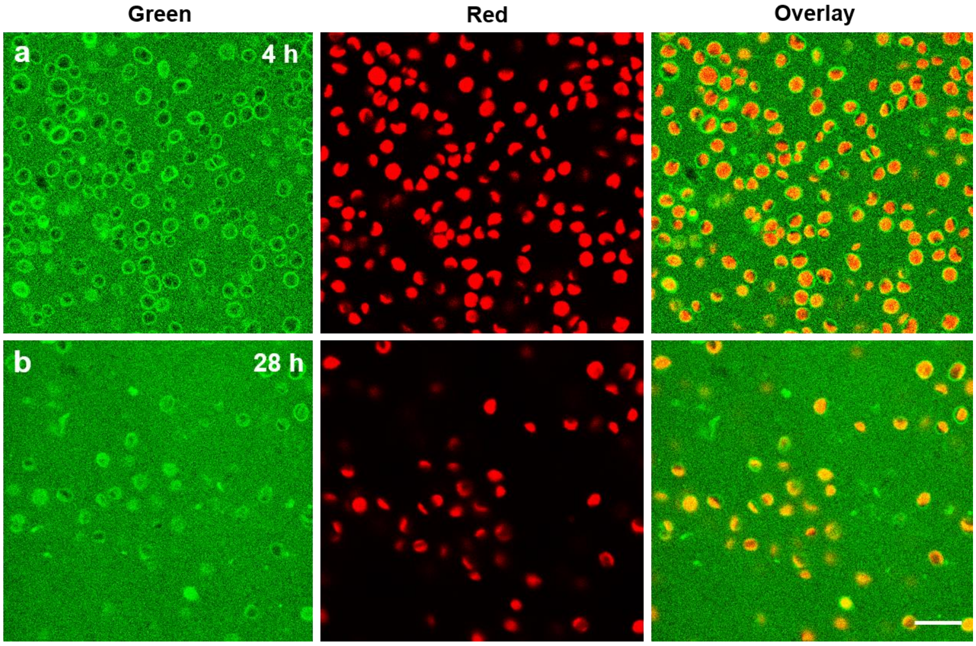

Supplementary Figure 20. Biomacromolecule uptake analysis for native *Chlorella* cells. Time-dependent confocal fluorescence microscopy images of native *Chlorella* cells dispersed in aqueous solution of the protein BSA for 4 (a) and 28 (b) hours. The algal cells are cultivated in sodium ascorbate-containing TAP culture medium. Green fluorescence is from FITC-labelled BSA and red fluorescence from intracellular chlorophyll of algal cells. Scale bars, 10 μm. All relevant experiments were performed independently at least three times with similar results. BSA permeates into the native *Chlorella* cells within 28 hours.

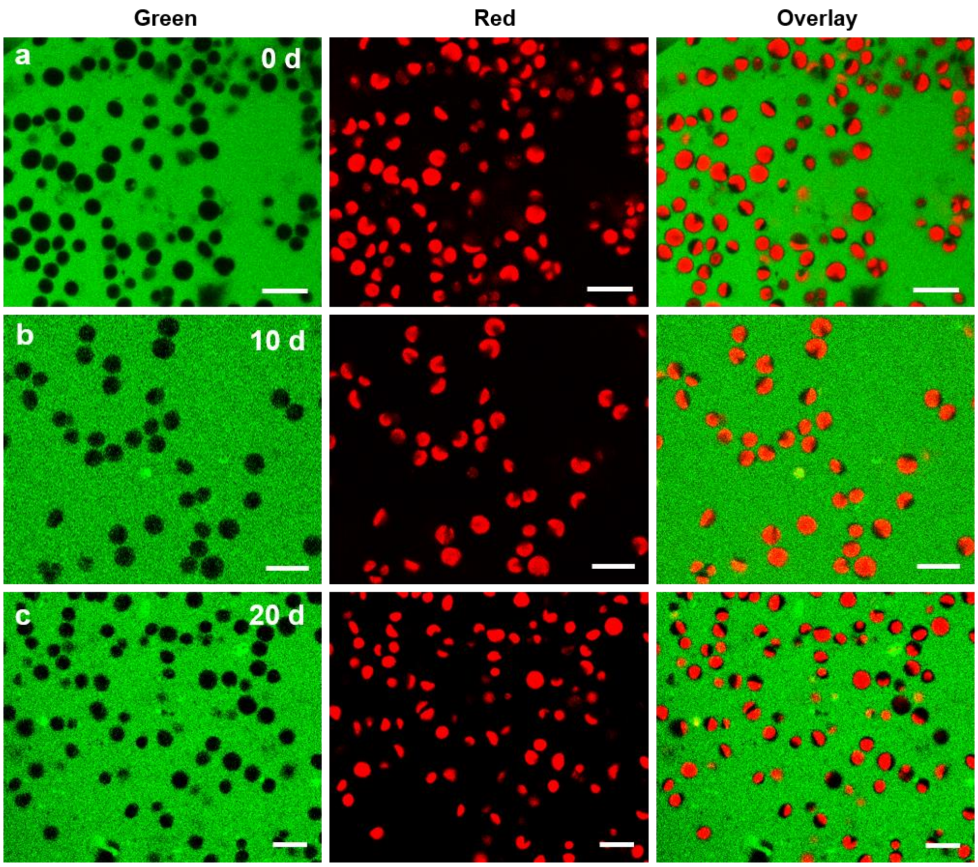

Supplementary Figure 21. Biomacromolecule uptake analysis for PPy/$CaCO_3$-coated *Chlorella* cells. Time-dependent confocal fluorescence microscopy images of PPy/$CaCO_3$-coated *Chlorella* cells dispersed in an aqueous solution of BSA for 0 (a), 10 (b) and 20 (c) days. The algal cells are cultivated in sodium ascorbate-containing TAP culture medium. Green fluorescence is from FITC labelled BSA and red fluorescence from intracellular chlorophyll of algal cells. Scale bars, 10 μm. All relevant experiments were performed independently at least three times with similar results. BSA macromolecules are excluded from the algal cell wall even after 20 days, demonstrating the decreased permeability of the PPy/$CaCO_3$ layer to biomacromolecules.

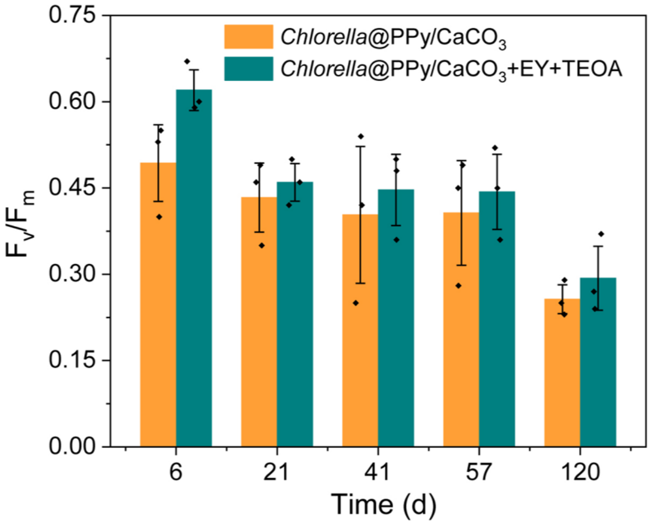

Supplementary Figure 22. Time-dependent measurements of F_v/F_m for PPy/$CaCO_3$-coated *Chlorella* cells with or without the addition of EY and TEOA. Samples are cultivated in sealed vials with sodium ascorbate-containing TAP culture medium and exposed to the daylight with an intensity of 65 μE m^{-2} s^{-1}. Data are presented as mean values ± SD, error bars indicate standard deviations (n = 3, biologically independent samples). Source data are provided as a Source Data file.

Supplementary Table 1. Hydrogen production performance comparison of algae by means of different methods.

Strains	Cultivation	H_2 production rate	Duration	Ref.
Chlamydomonas reinhardtii (mutant strain pgr5)	Sulfur free conditions	3.35 μmol H_2 (mg chlorophyll)$^{-1}$ h^{-1}	192 hours	1
Chlamydomonas reinhardtii CC124	Sulfur free conditions	0.93 μmol H_2 (mg chlorophyll)$^{-1}$ h^{-1}	192 hours	1
Chlorella pyrenoidosa	TAP culture medium, supplied with DMSO	0.65 μmol H_2 (mg chlorophyll)$^{-1}$ h^{-1}	48 hours	2
Chlorella pyrenoidosa	TAP culture medium	0.35 μmol H_2 (mg chlorophyll)$^{-1}$ h^{-1}	48 hours	3
Chlorella pyrenoidosa	TAP culture medium	0.32 μmol H_2 (mg chlorophyll)$^{-1}$ h^{-1}	168 hours	4
Chlorella pyrenoidosa	TAP culture medium	0.43 μmol H_2 (mg chlorophyll)$^{-1}$ h^{-1}	72 hours	5
Chlamydomonas reinhardtii	TAP culture medium	0.44 μmol H_2 (mg chlorophyll)$^{-1}$ h^{-1}	624 hours	6
Chlorella pyrenoidosa	TAP culture medium	0.37 μmol H_2 (mg chlorophyll)$^{-1}$ h^{-1}	264 hours	7
Platymonas subcordiformis	Two-stage incubation, sulfur free system supplied with CCCP	1.92 μmol H_2 (mg chlorophyll)$^{-1}$ h^{-1}	12 hours	8
Synechocystis PCC 6803	BG-11 culture medium	6.55 μmol H_2 (mg chlorophyll)$^{-1}$ h^{-1}	144 hours	9
Chlorella pyrenoidosa	TAP culture medium	4.43 μmol H_2 (mg chlorophyll)$^{-1}$ h^{-1}	72 days	This work

Supplementary Table 2. Chlorophyll fluorescence characteristics of PPy/$CaCO_3$-coated *Chlorella* cells with or without the addition of EY and TEOA. In both cases, the algal cells were cultivated in sodium ascorbate-containing TAP culture medium.

Parameter	Definition	Ratio[a]
φ_{Eo}	quantum yield for electron transport	1.16
S_m	energy required to completely reduce Q_A, namely the size of PQ pool on the receptor side of PSII reaction center	1.04
ABS/CS_m	absorbed energy per excited cross section	1.05
ET_o/CS_m	energy used for electron transfer per excited cross section	1.22
TR_o/CS_m	trapped energy per excited cross section	1.13
RE_o/CS_m	energy used for reducing end electron acceptors per excited cross section	1.29
RC/CS_m	density of PSII reducing centres per excited cross section	1.31
PI_{abs}	performance index based on absorption basis	1.57
PI_{cs}	performance index based on cross section basis	1.65
PI_{total}	performance index for energy conversion from photons absorbed by PSII to reduction of PSI and acceptors	1.66

[a] Ratio of photosynthetic parameters of the PPy/$CaCO_3$-coated *Chlorella* cell with and without the addition of EY and TEOA

Supplementary Note 1. Mechanism of Fe(III)-mediated oxidation of ascorbate

Fe(III)-doped PPy layer on algal cells catalyses the oxidation of ascorbate in the solution, which results in the oxygen depletion process. The mechanism is given below[10]:

$$A + 2Fe(III) \rightarrow DHA + 2Fe(II) + 2H^+$$
$$2Fe(II) + 2O_2 \rightarrow 2Fe(III) + 2O_2^-$$
$$2O_2^- + 2H^+ + Fe(III) \rightarrow O_2 + H_2O_2 + Fe(III)$$
$$H_2O_2 + Fe(III)\text{-}A \rightarrow Fe(III)\text{-}DHA + 2H_2O$$
$$\text{Overall, } A + 1/2O_2 \rightarrow DHA + H_2O$$

where A is ascorbate, and DHA was the dehydroascorbic acid.

Supplementary Note 2. Calculation of the light-to-hydrogen efficiency

The efficiency of the conversion of light energy to hydrogen energy is calculated by supplementary Eq. (**1**):

$$\eta\ (\%) = \frac{(\Delta G°\text{-}RTln(P°/P))R_{\text{H}}}{E_{\text{s}}A} * 100 \quad \textbf{(1)}$$

where, ΔG^{o} is the standard Gibbs free energy of hydrogen (237200 J mol^{-1} at 25 ºC), R is the universal gas content, T is the absolute temperature, P^{o} and P are the standard and observed hydrogen pressures (atm), R_{H} is the hydrogen production rate (mol s^{-1}), E_{S} is the energy of the incident light radiation (averaged over 400-700 nm; 1 μE = 0.214 J, the energy of 1 mol of 560 nm photons; illumination from one side), A is the surface area.

Supplementary Note 3. Calculation of the chlorophyll fluorescence parameters

F_v is calculated by the supplementary Eq. (**2**):

$$F_{\text{v}} = F_{\text{m}}\text{-}F_{\text{o}} \quad \textbf{(2)}$$

φ_{Eo} is calculated by the supplementary Eq. (**3**) and (**4**):

$$V_{\text{J}} = (F_{2\text{ ms}}\text{-}F_{\text{o}})/(F_{\text{m}}\text{-}F_{\text{o}}) \quad \textbf{(3)}$$

$$\varphi_{Eo} = (F_v/F_m)\ (1\text{-}V_J) \quad \mathbf{(4)}$$

S_m, ABS/CS_m and ET_o/CS_m are calculated by the supplementary Eq. (**5**), (**6**) and (**7**), respectively:

$$S_m = Area/(F_m\text{-}F_o) \quad \mathbf{(5)}$$

$$ABS/CS_m = F_m \quad \mathbf{(6)}$$

$$ET_o/CS_m = (ABS/CS_m)\ \varphi_{Eo} \quad (7)$$

TR_o/CS_m is calculated by the supplementary Eq. (**8**) and (**9**):

$$\varphi_{Po} = F_v/F_m \quad \mathbf{(8)}$$

$$TR_o/CS_m = (ABS/CS_m)\ \varphi_{Po} \quad (9)$$

RE_o/CS_m is calculated by the supplementary Eq. (**10**), (**11**) and (**12**):

$$V_I = (F_{30\,ms}\text{-}F_o)/(F_m\text{-}F_o) \quad \mathbf{(10)}$$

$$\delta_{Ro} = (1\text{-}V_J)\ (1\text{-}V_I) \quad \mathbf{(11)}$$

$$RE_o/CS_m = (ET_o/CS_m)\ \delta_{Ro} \quad \mathbf{(12)}$$

RC/CS_m is calculated by the supplementary Eq. (**13**):

$$RC/CS_m = \varphi_{Po}\ (ABS/CS_m)\ (V_J/M_o) \quad \mathbf{(13)}$$

PI_{abs} is calculated by the supplementary Eq. (**14**), (**15**) and (**16**):

$$RC/ABS = M_o/V_J/\varphi_{Po} \quad (14)$$

$$\psi_{Eo} = 1\text{-}V_J \quad \mathbf{(15)}$$

$$PI_{abs} = (RC/ABS)\ (\varphi_{Po}/(1\text{-}\varphi_{Po}))\ (\psi_{Eo}/(1\text{-}\psi_{Eo})) \quad \mathbf{(16)}$$

PI_{cs} is calculated by the supplementary Eq. (**17**):

$$PI_{cs} = (RC/CS_m)\ (\varphi_{Po}/(1\text{-}\varphi_{Po}))\ (\psi_{Eo}/(1\text{-}\psi_{Eo})) \quad \mathbf{(17)}$$

PI_{total} is calculated by the supplementary Eq. (**18**):

$$PI_{total} = (RC/ABS)\ (\varphi_{Po}/(1\text{-}\varphi_{Po}))\ (\psi_{Eo}/(1\text{-}\psi_{Eo}))\ (\delta_{Ro}/(1\text{-}\delta_{Ro})) \quad \mathbf{(18)}$$

Supplementary references

1. Khosravitabar F, Hippler M. A new approach for improving microalgal biohydrogen photoproduction based on safe & fast oxygen consumption. *Int. J. Hydrogen Energy* **44,** 17835-17844 (2019).
2. Shu L, Xiong W, Shao C, Huang T, Duan P, Liu K*, et al.* Improvement in the photobiological hydrogen production of aggregated *Chlorella* by dimethyl sulfoxide. *ChemBioChem* **19,** 669-673 (2018).
3. Xiong W, Zhao X, Zhu G, Shao C, Li Y, Ma W*, et al.* Silicification-induced cell aggregation for the sustainable production of H_2 under aerobic conditions. *Angew. Chem. Int. Ed.* **54,** 11961-11965 (2015).
4. Su D, Qi J, Liu X, Wang L, Zhang H, Xie H*, et al.* Enzyme-modulated anaerobic encapsulation of *Chlorella* cells allows switching from O_2 to H_2 production. *Angew. Chem. Int. Ed.* **58,** 3992-3995 (2019).
5. Xu Z, Wang S, Zhao C, Li S, Liu X, Wang L*, et al.* Photosynthetic hydrogen production by droplet-based microbial micro-reactors under aerobic conditions. *Nat. Commun.* **11,** 5985 (2020).
6. Chen J, Li J, Li Q, Wang S, Wang L, Liu H*, et al.* Engineering a chemoenzymatic cascade for sustainable photobiological hydrogen production with green algae. *Energy Environ. Sci.* **13,** 2064-2068 (2020).
7. Chen J, Li Y, Li M, Shi J, Wang L, Luo S*, et al.* Chemical flocculation-based green algae materials for photobiological hydrogen production. *ACS Appl. Bio. Mater.* **5,** 897-903 (2022).
8. Ran C, Yu X, Jin M, Zhang W. Role of carbonyl cyanide m-chlorophenylhydrazone in enhancing photobiological hydrogen production by marine green alga platymonas subcordiformis. *Biotechnol. Prog.* **22,** 438-443 (2006).
9. Touloupakis E, Benavides AMS, Cicchi B, Torzillo G. Growth and hydrogen production of outdoor cultures of *Synechocystis* PCC 6803. *Algal Res.* **18,** 78-85 (2016).
10. Khosravitabar F, Hippler M. A new approach for improving microalgal biohydrogen photoproduction based on safe & fast oxygen consumption. *Int. J. Hydrogen Energy* **44**, 17835-17844 (2019).
