# Supplementary material for: Algal cell bionics as a step towards photosynthesis-independent hydrogen production
Source: Nat Commun. 2023 Apr 4;14:1872. doi: 10.1038/s41467-023-37608-4 (PMC10073198; doi:10.1038/s41467-023-37608-4)
Supplement: Supplementary file 3 — Reporting Summary [file 41467_2023_37608_MOESM3_ESM.pdf]

nature portfolio

Corresponding author(s): Stephen Mann, Xin Huang

Last updated by author(s): Mar 11, 2023

Reporting Summary

Nature Portfolio wishes to improve the reproducibility of the work that we publish. This form provides structure for consistency and transparency in reporting. For further information on Nature Portfolio policies, see our Editorial Policies and the Editorial Policy Checklist.

Statistics

For all statistical analyses, confirm that the following items are present in the figure legend, table legend, main text, or Methods section.

n/a	Confirmed	
☐	☒	The exact sample size (*n*) for each experimental group/condition, given as a discrete number and unit of measurement
☐	☒	A statement on whether measurements were taken from distinct samples or whether the same sample was measured repeatedly
☒	☐	The statistical test(s) used AND whether they are one- or two-sided<br>*Only common tests should be described solely by name; describe more complex techniques in the Methods section.*
☒	☐	A description of all covariates tested
☐	☒	A description of any assumptions or corrections, such as tests of normality and adjustment for multiple comparisons
☐	☒	A full description of the statistical parameters including central tendency (e.g. means) or other basic estimates (e.g. regression coefficient) AND variation (e.g. standard deviation) or associated estimates of uncertainty (e.g. confidence intervals)
☒	☐	For null hypothesis testing, the test statistic (e.g. *F*, *t*, *r*) with confidence intervals, effect sizes, degrees of freedom and *P* value noted<br>*Give P values as exact values whenever suitable.*
☒	☐	For Bayesian analysis, information on the choice of priors and Markov chain Monte Carlo settings
☐	☒	For hierarchical and complex designs, identification of the appropriate level for tests and full reporting of outcomes
☒	☐	Estimates of effect sizes (e.g. Cohen's *d*, Pearson's *r*), indicating how they were calculated

Our web collection on statistics for biologists contains articles on many of the points above.

Software and code

Policy information about availability of computer code

Data collection	X-ray diffraction (XRD) patterns were acquired using a D/MAX-RC thin-film X-ray diffractometer equipped with a nickel filter. Scanning electron microscopy (SEM) images were acquired on a SU8000 instrument with the samples sputter-coated with 10 nm platinum, equipped with an energy-dispersive spectroscope. The pH measurements were conducted with a Seven Compact meter (METTLER TOLEDO, SUI). Transmission electron microscopy (TEM) images were obtained on a JEM-1400, with a filament at 120kV under bright field mode. Fourier Transform infrared spectroscopy (FTIR) measurements were performed on PerkinElmer spectrometer with a $LiTaO_3$ detector (Spectrum Two, USA). UV-vis spectra were measured on a PerkinElmer spectrophotometer (Lambda 750S, USA). Confocal scanning laser microscopy (CSLM) images were obtained on a Leica SP8 confocal laser scanning microscope attached to a Leica DMI 6000 inverted epifluorescence microscope. The dissolved oxygen content was detected using a dissolved oxygen meter (METTLER TOLEDO, F4-Field). the chlorophyll fluorescence kinetics were conducted using chlorophyll fluorometer (Yaxin, 1161G, Beijing, China). the CV data were conducted on the electrochemical workstation (Chenhua CHI604E, Shanghai, China). The hydrogen data were collected using a Hydrogen Detector (AP-B-H2-F). The light intensity as well as the spectra were collected using a light analyzer (OHSP-350P).
Data analysis	The AFM results analyses were conducted using NanoScope Analysis 1.5. The counting of cell numbers was carried out using Image J 1.53e. The XPS peak separation was performed by XPSPEAK41. Data process was conducted using Origin 2021.

For manuscripts utilizing custom algorithms or software that are central to the research but not yet described in published literature, software must be made available to editors and reviewers. We strongly encourage code deposition in a community repository (e.g. GitHub). See the Nature Portfolio guidelines for submitting code & software for further information.

Data

Policy information about availability of data

All manuscripts must include a data availability statement. This statement should provide the following information, where applicable:

- Accession codes, unique identifiers, or web links for publicly available datasets
- A description of any restrictions on data availability
- For clinical datasets or third party data, please ensure that the statement adheres to our policy

The authors declare that all relevant data supporting the finding of this study are available within this paper and its Supplementary Information files. Additional data are available from the corresponding author upon reasonable request.

Human research participants

Policy information about studies involving human research participants and Sex and Gender in Research.

Reporting on sex and gender	Not applicable in this study.
Population characteristics	Not applicable in this study.
Recruitment	Not applicable in this study.
Ethics oversight	Not applicable in this study.

Note that full information on the approval of the study protocol must also be provided in the manuscript.

Field-specific reporting

Please select the one below that is the best fit for your research. If you are not sure, read the appropriate sections before making your selection.

☒ Life sciences ☐ Behavioural & social sciences ☐ Ecological, evolutionary & environmental sciences

For a reference copy of the document with all sections, see nature.com/documents/nr-reporting-summary-flat.pdf

Life sciences study design

All studies must disclose on these points even when the disclosure is negative.

Sample size	No sample size calculation was performed and they are described in the figure legend (at least three replicates).
Data exclusions	No data were excluded from the analyses.
Replication	Each experiment was performed at more than three independent replications and was described in the figure legend.
Randomization	Algal cells were randomly grouped for operation. Cells samples were randomly grouped for experiments. Enough replications and control groups were carried out in consistent environment to control covariates.
Blinding	Blinding is not possible in this study.

Reporting for specific materials, systems and methods

We require information from authors about some types of materials, experimental systems and methods used in many studies. Here, indicate whether each material, system or method listed is relevant to your study. If you are not sure if a list item applies to your research, read the appropriate section before selecting a response.

Materials & experimental systems

n/a	Involved in the study
☐	☒ Antibodies
☒	☐ Eukaryotic cell lines
☒	☐ Palaeontology and archaeology
☒	☐ Animals and other organisms
☒	☐ Clinical data
☒	☐ Dual use research of concern

Methods

n/a	Involved in the study
☒	☐ ChIP-seq
☒	☐ Flow cytometry
☒	☐ MRI-based neuroimaging

Antibodies

Antibodies used

Anti-hydrogenase A (Agrisera) and goat anti-rabbit IgG (H&L)-HRP conjugate (Agrisera) were used as the primary antibody and secondary antibody, respectively.

Validation

the validations of Anti-hydrogenase A and goat anti-rabbit IgG (H&L)-HRP conjugate could be referred to the following websites: https://www.agrisera.com/en/artiklar/hyda-iron-hydrogenase-hyda1_hyda2.html (Anti-hydrogenase A, primary antibody); https://www.agrisera.com/en/artiklar/goat-anti-rabbit-igg-hl.html (goat anti-rabbit IgG (H&L)-HRP conjugate, secondary antibody).
